# Natalia Shustova answers questions about 15 years of research on covalent organic frameworks

**DOI:** 10.1038/s41467-020-19299-3

**Published:** 2020-11-16

## Abstract

Prof. Natalia Shustova is the Peter and Bonnie McCausland Associate Professor at the University of South Carolina. After receiving her Ph.D. in Physical Chemistry from the Moscow State University and a second Ph.D. in Inorganic Chemistry from the Colorado State University, she became interested in porous frameworks during her time as a Postdoctoral Associate in the group of Prof. Mircea Dincă at MIT. After starting her independent research career first as an Assistant Professor at the University of South Carolina in 2013 and since 2017 as Associate Professor, her research interest lies in the development of materials for sustainable energy conversion, sensing and artificial biomimetic systems.

Tell us a little bit about your research background and how you became interested in the chemistry of porous materials and covalent organic frameworks.

I started my education and scientific career as a materials scientist at Moscow State University. During the work pursuing my two Ph.D. degrees in inorganic and physical chemistry I became a molecular synthetic chemist with an interest in organic electron acceptors. Versatile highly crystalline frameworks such as covalent organic frameworks (COFs) or metal organic frameworks (MOFs) provided me a unique opportunity to merge all the directions from my background in physical and inorganic chemistry with materials science in the pursuit of materials discovery and development. My journey back then in the field of porous frameworks started at MIT where I had my post-doctoral training in the research group of Prof. Mircea Dincă. Initially, the main attraction which was drawing me to the field of COFs were the unlimited opportunities that I believe this platform can provide for the exploration of synthesis, physical properties, and even materials chemistry. Lately, I have been inspired by the possible advances that the porous frameworks can bring to multiple areas ranging from oxygen evolution to nuclear waste administration in order to provide valuable potential materials to expand the technological landscape. Furthermore, I believe that the field of porous frameworks is a strong magnet for multidisciplinary collaborations that usually result in an unusual break-through. This tendency can also be clearly seen in the latest publications.

Looking back on 15 years research on COFs, what were the highlights in COF research and which expectations remained unfulfilled?

Over the past decade, great efforts have been pursued in the development and the advancement of the COF field. The chemistry and synthetic routes for COF preparation have been delineated which leads to a rapid progress in the field. However, this rapid development also brings the necessity for standardized nomenclature to catalogue these innovative frameworks (COFs) into distinct classes that has not been developed so far. Overall, COFs are a powerful platform that can advance a myriad of applications, extending from gas storage and separation to catalysis. In contrast to zeolites, that have limited intrinsic porosity, and amorphous porous polymers, that lack a clear structure–property correlation, COFs are very attractive candidates for pollution extraction or water purification. Moreover, COFs have a great potential in catalysis through tuneable pore environment and straightforward wall decoration with multifunctional catalytic active sites that we actually investigate in my group. Another field for applications of COFs is sensing, in which the utilization of the well-defined organic scaffold can lead to rapid signal transduction and, as I believe, signal amplification. The last, but not least, application that I want to highlight is the development of crystalline organic semiconductors possessing high conductivity and mobility, which has recently surfaced as a new emerging area of research.

In your opinion, which challenges need to be tackled in order to move the field further?

Despite tremendous effort and success, there are still challenges in the field of chemistry, physics and materials science in relation to COFs that must be resolved. For instance the development of a general method for single crystal growth is highly desirable for the establishment of fundamental aspects related to structure–property correlation and of course theoretical modelling. Another aspect that requires attention from the scientific community is the development of new synthetic routes that will further broaden the diversity of COF motives and advance the field of materials science while at the same time—and this is very important—leading to crystalline materials. From the other aspect, the understanding of interactions between photons or electrons and a COF crystalline skeleton occurring in one layer, in multiple layers, or in the bulk material is the key for a number of applications including highly porous electrodes and sensors. Biocompatible COFs is another intriguing direction for research that currently lacks extensive studies of framework toxicity and stability.

In summary, I believe that we have only touched the surface of possible options of synthetic methodologies for the preparation of porous crystalline purely organic frameworks such as COFs. However, crucial cornerstones such as thermodynamics, electronics, photosynthesis, and theoretical modelling are still under heavy and exciting development from my point of view.

What needs to be done in order to make COFs more widely adopted and to get a foothold in industry and industrial applications?

Since the beginning of COFs there have been a significant success in finding novel topologies and organic linker engineering resulting in tailoring the intrinsic properties of COFs, but their widespread implementation in device components has not been accomplished yet. This requires overcoming challenges to achieve low cost, high throughput preparation and processability of COFs for mass production. For instance, the preparation of high quality thin films of COFs with controllable thickness is a main challenge in COF research. Moreover, the ability to integrate a COF into a device is a difficult task and needs a multidisciplinary approach and input from materials development and device fabrication.

Where do you see the field going next?

I hope until this point, I have highlighted the most important features of COFs for a wide range of applications that not only span the classical applications known for metal-organic frameworks such as gas storage and separation but also recognizes a shift towards the less studied areas of optoelectronics or waste management. COF-based materials display an enormous potential especially since the movement towards tailored COF design for specific applications. For instance, development of COF materials with dynamically controlled photophysical behaviour has recently taken off. The advent of novel synthetic approaches, unique growth techniques, and optimized device fabrication methods for COF-based materials is anticipated. And these areas in COF research will be drastically expanded within the next years. However, I strongly believe that this should be accompanied with a growing interest towards a refinement of design principles and fundamental mechanistic studies of physical processes in COF-based materials in order to benefit from their evolving technological possibilities. Therefore, the advances highlighted here are a very promising start but also show a compelling future for COF research.

*The interview was conducted by Senior Editor Dr. Johannes Kreutzer*.

